# Development of Opsonic Mouse Monoclonal Antibodies against Multidrug-Resistant Enterococci

**DOI:** 10.1128/IAI.00276-19

**Published:** 2019-08-21

**Authors:** Ermioni Kalfopoulou, Diana Laverde, Karmela Miklic, Felipe Romero-Saavedra, Suzana Malic, Filippo Carboni, Roberto Adamo, Tihana Lenac Rovis, Stipan Jonjic, Johannes Huebner

**Affiliations:** a Division of Paediatric Infectious Diseases, Dr. von Hauner Children’s Hospital, Ludwig Maximilians University, Munich, Germany; b Center for Proteomics, Faculty of Medicine, University of Rijeka, Rijeka, Croatia; c GSK, Siena, Italy; University of Michigan-Ann Arbor

**Keywords:** DHG, *Enterococcus faecalis*, *Enterococcus faecium*, SagA, capsular polysaccharide, diheteroglycan, hybridoma technology, monoclonal antibodies, opsonic, opsonophagocytic assay

## Abstract

Multidrug-resistant enterococci are major causes of hospital-acquired infections. Immunotherapy with monoclonal antibodies (MAbs) targeting bacterial antigens would be a valuable treatment option in this setting. Here, we describe the development of two MAbs through hybridoma technology that target antigens from the most clinically relevant enterococcal species. Diheteroglycan (DHG), a well-characterized capsular polysaccharide of Enterococcus faecalis, and the secreted antigen A (SagA), an immunogenic protein from Enterococcus faecium, are both immunogens that have been proven to raise opsonic and cross-reactive antibodies against enterococcal strains. For this purpose, a conjugated form of the native DHG with SagA was used to raise the antibodies in mice, while enzyme-linked immunosorbent assay and opsonophagocytic assay were combined in the selection process of hybridoma cells producing immunoreactive and opsonic antibodies targeting the selected antigens. From this process, two highly specific IgG1(κ) MAbs were obtained, one against the polysaccharide (DHG.01) and one against the protein (SagA.01). Both MAbs exhibited good opsonic killing against the target bacterial strains: DHG.01 showed 90% killing against E. faecalis type 2, and SagA.01 showed 40% killing against E. faecium 11231/6. In addition, both MAbs showed cross-reactivity toward other E. faecalis and E. faecium strains. The sequences from the variable regions of the heavy and light chains were reconstructed in expression vectors, and the activity of the MAbs upon expression in eukaryotic cells was confirmed with the same immunological assays. In summary, we identified two opsonic MAbs against enterococci which could be used for therapeutic or prophylactic approaches against enterococcal infections.

## Abstract (precis)

Multidrug-resistant enterococci are major causes of hospital-acquired infections. Immunotherapy with monoclonal antibodies (MAbs) targeting bacterial antigens would be a valuable treatment option in this setting. Here, we describe the development of two MAbs through hybridoma technology that target antigens from the most clinically relevant enterococcal species.

## INTRODUCTION

Multiresistant Gram-positive bacteria such as enterococci are major causes of hospital-acquired infections. The two most prevalent enterococcal species associated with high incidences of nosocomial infections are Enterococcus faecium and Enterococcus faecalis (1, 2). Besides their intrinsic antibiotic resistance to aminoglycosides and β-lactams, resistance to vancomycin as well as to newer antibiotics is of clinical concern (3, 4). With the emergence of these multidrug-resistant bacteria and their prevalence in the clinical setting, passive immunotherapy is a promising treatment option (5). Passive immunotherapy against infectious diseases is limited to a small number of FDA-licensed monoclonal antibodies (MAbs) but remains an emerging field with many promising candidates to address these health threats (6). One of the main challenges in MAb production is their lack of broad coverage, which is caused by their high specificity and the antigenic variability of the pathogens, even in the same bacterial species (7). For this purpose several protein and polysaccharide targets have been explored in enterococci for the development of passive immunotherapy regimens, although no direct comparison between these targets exists so far (5, 8–12).

E. faecalis strains have been grouped into four serotypes, CPS-A to -D, by immunological and genetic methods (13). McBride et al. evaluated the genetic diversity of E. faecalis strains and showed that about half of CPS-C strains were more virulent than CPS-A and -B strains (14). Serotypes CPS-C and CPS-D possess a capsular polysaccharide which is solely presented in these strains and characterizes their surface composition and serological recognition compared to those of serotypes CPS-A and CPS-B. This immunogenic capsular polysaccharide, diheteroglycan (DHG), was identified by Pazur et al. and structurally elucidated by Theilacker et al. and Krylov et al. (12, 15, 16). We have previously shown that rabbit serum raised against DHG (anti-DHG) mediates opsonophagocytic killing (OPK) of the encapsulated strains and promotes bacterial clearance in infected mice by reducing the bacterial load in livers and kidneys (12). It was also suggested that passive immunotherapy against DHG could provide protection against encapsulated E. faecalis strains (12).

In contrast to E. faecalis, the capsular polysaccharide composition of E. faecium strains has not been extensively explored. However, several cell surface-associated protein antigens have been identified (10, 17, 18). Secreted antigen A (SagA), initially characterized by Teng et al., has been shown to bind to extracellular matrix proteins and to be a major component of the biofilm matrix of E. faecium (19, 20). We have demonstrated that SagA induces opsonic and protective antibodies against all vancomycin-resistant E. faecium strains tested, suggesting that a MAb targeting SagA could serve as a promising candidate for therapeutic intervention (10, 18). In addition, our results support the use of SagA as a vaccine target against nosocomial E. faecium strains and potentially as a carrier protein in glycoconjugated vaccine formulations (12, 14, 56).

To generate high-affinity MAbs against these two immunogens, hybridoma technology was used. This technique was initially introduced in 1975 by Köhler and Milstein after fusion of myeloma cell lines with antibody-secreting B cells (21). Since then, this technique has been widely applied in the generation of murine MAbs against pathogens targeting polysaccharide and protein antigens (22–26). Polysaccharides are poorly immunogenic and are usually incapable of triggering a T cell-dependent immune response (27). Conjugation of polysaccharides with a carrier protein overcomes the obstacle of low immunogenicity by provoking T cell-dependent immune responses (27). This method has numerous implementations in the production of glycoconjugate vaccines and also as immunogens for the production of polysaccharide-specific MAbs in mice (23, 24, 28).

In this study, we developed, purified, and characterized two mouse MAbs against enterococci, one specific to the capsular polysaccharide DHG and another one toward the protein SagA. Moreover, we exploited the immunogenicity of SagA and used it not only as an E. faecium-specific antigen but also as a carrier protein for a glycoconjugate with DHG. The MAbs obtained upon immunization of mice with this glycoconjugate specifically recognized these two antigens, targeting either E. faecium or E. faecalis strains, and mediated *in vitro* OPK of the respective strains.

## RESULTS

### Generation of highly specific antibodies against DHG and SagA in mice.

**FIG 1:**
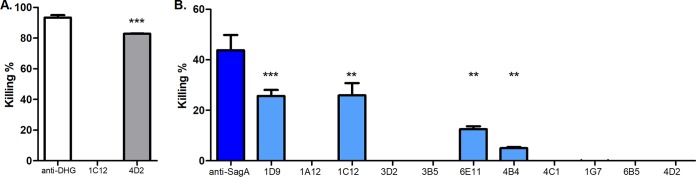
Opsonophagocytic killing activity of supernatants from the mother-wells of the hybridomas against E. faecalis type 2 and E. faecium 11231/6. The opsonophagocytic killing activity of the supernatants from the mother-wells was evaluated against E. faecalis type 2 (A) and E. faecium 11231/6 (B). The bars of the evaluated supernatants specific to DHG and to SagA are represented with gray and light blue color, respectively. Polyclonal sera raised in rabbits against the native polysaccharide DHG (white) and SagA (dark blue) were used as positive controls at 25 μg/ml and 200 μg/ml, respectively. Bars represent mean data, and the error bars represent the standard errors of the means. *, *P* < 0.05; **, *P* < 0.01; ***, *P* < 0.001; all by Student's *t* test against the supernatants of 1C12 (A) and 4D2 (B). Results are representative of at least two independent experiments.

**FIG 2:**
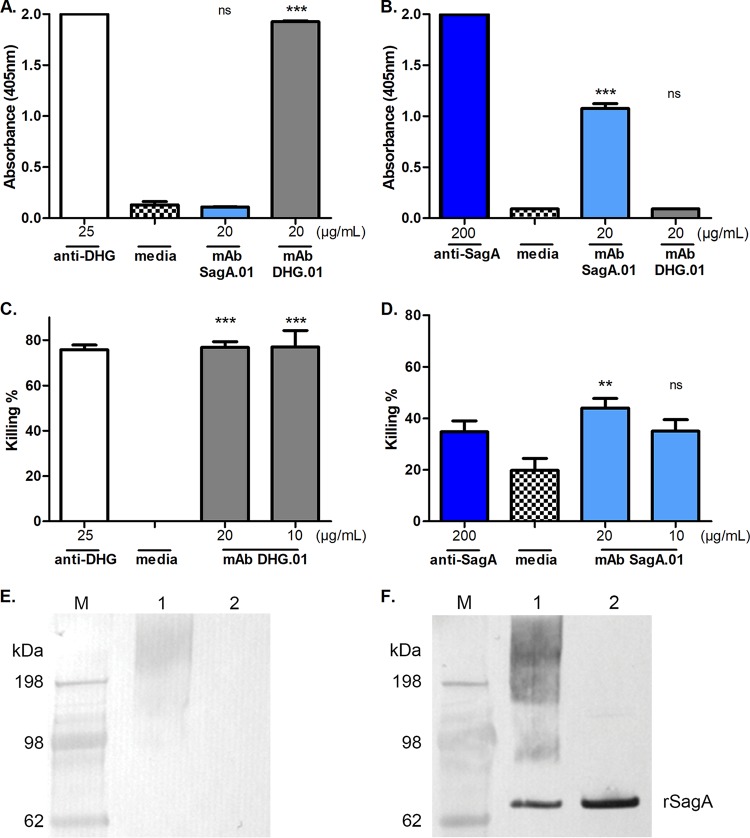
Specificity and opsonophagocytic killing activity of the supernatants from the hybridoma cells producing MAbs DHG.01 and SagA.01 against E. faecalis type 2 and E. faecium 11231/6, respectively. The binding of the MAbs DHG.01 (gray) and SagA.01 (light blue) was evaluated by ELISA against the native DHG (A) and rSagA (B). The opsonophagocytic killing activity of the supernatants from the hybridoma cells producing MAb DHG.01 (gray) and SagA.01 (light blue) at different dilutions was evaluated against E. faecalis type 2 (C) and E. faecium 11231/6 (D), respectively. Polyclonal sera raised in rabbits against the native polysaccharide DHG (white) and SagA (dark blue) were used as positive controls. Hybridoma cell culture medium was used as a negative control. Bars represent mean data, and the error bars represent the standard errors of the means. ns (not significant), *P*  ≥ 0.05; *, *P* < 0.05; **; *P* < 0.01; ***, *P* < 0.001; all by one-way ANOVA with Dunnett's multiple comparison to the negative control (media). The antibody specificity was confirmed by Western blotting against DHG-SagA (lanes 1) and unconjugated SagA (lanes 2) using the supernatants from the hybridomas expressing MAbs DHG.01 (E) and SagA.01 (F). SeeBlue Plus2 prestained protein standard (M) was used to assess the molecular weight of the samples, e.g., rSagA is around 55 kDa. Results are representative of at least two independent experiments.

Mice were immunized using DHG-SagA and Freund's incomplete adjuvant. The mouse with the highest titers against the glycoconjugate was sacrificed, and its splenocytes were fused with SP2/O myeloma cells. After the fusion, the original unstable hybrid cells (termed mother-wells) and their subsequent clones were selected by enzyme-linked immunosorbent assay (ELISA) and opsonophagocytic assay (OPA). ELISA was performed in order to obtain highly specific clones either to the protein SagA, which in this case had a dual role as the carrier protein and as the immunogen, or to the polysaccharide DHG. The supernatants from the clones were double screened in ELISA against SagA and DHG in order to obtain MAbs that were highly specific toward one of the two antigen targets and that exhibit no cross-reactivity to the other immunogen. Mother-wells with a mean *A*_492_/*A*_630_ higher than 0.6 absorbance units in ELISA against the target immunogen (e.g., DHG) and less than 0.3 absorbance units to the remaining antigen (SagA in this case) were considered specific to the target immunogen and were retained as potential candidates for cloning, whereas the rest of the mother-wells were excluded. Through this method we identified one mother-well, 4D2, with specificity to DHG and ten mother-wells (1D9, 1A12, 1C12, 3D2, 3B5, 6E11, 4B4, 4C1, 1G7, and 6B5) that produced antibodies with high immunoreactivity toward SagA (data not shown). OPA then was used as a selection method in order to identify the mother-wells that were producing opsonic antibodies. The mother-well 4D2 produced antibodies with high opsonic killing against E. faecalis type 2 but no opsonic killing against E. faecium 11231/6 (Fig. 1A and B). On the contrary, the mother-well 1C12 exhibited high opsonic killing against the evaluated E. faecium 11231/6 strain but no opsonic killing against E. faecalis type 2 (Fig. 1A and B). Therefore, the mother-wells 4D2 and 1C12 were subjected to cloning by limiting dilution and screening again by ELISA against DHG and SagA for the development of hybridoma cells targeting DHG and SagA, respectively. Three clones from each mother-well were stored, and the obtained MAbs, DHG.01 and SagA.01, exhibited high specificity in ELISA toward the targets while no cross-reactivity was observed (Fig. 2A and B). In order to assess whether the opsonophagocytic activity was retained, the supernatants from the hybridoma cells were tested again in OPA against the evaluated strains. Supporting and completing the selection process, the supernatants with DHG.01 and SagA.01, both at concentrations of 20 μg/ml and 10 μg/ml, exhibited high opsonic killing against E. faecalis type 2 (77%) and E. faecium 11231/6 (44 and 35%), respectively (Fig. 2C and D). MAb specificity was also proven by Western blotting, where DHG.01 recognized only the glycoconjugate DHG-SagA and not the unconjugated SagA (Fig. 2E). On the contrary, SagA.01 recognized the unconjugated protein and subsequently the glycoconjugate DHG-SagA (Fig. 2F). Finally, both antibodies were determined by ELISA to be IgG1 class for the heavy chain and kappa for the light chain.

### DHG.01 mediates opsonophagocytosis due to its binding to DHG.

**FIG 3:**
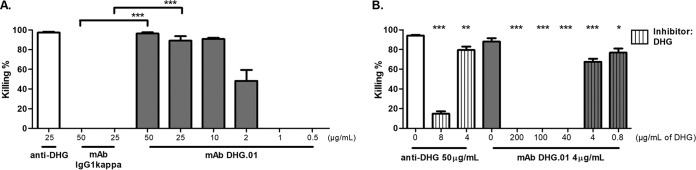
Opsonophagocytic killing activity of the purified MAb DHG.01 against E. faecalis type 2 (A) and inhibition of its opsonophagocytic killing activity using native DHG (B). (A) The opsonophagocytic killing activity of the MAb DHG.01 was evaluated at different dilutions, ranging from 50 μg/ml to 0.5 μg/ml (gray). Polyclonal serum raised in rabbits against the native polysaccharide DHG (anti-DHG) was used as a positive control (white), and a MAb of the same isotype, IgG1(κ), was used as a negative control. Bars represent mean data, and the error bars represent the standard errors of the means. ***, *P* < 0.001 by Student's *t* test against the negative control [MAb IgG1(κ)]. (B) The purified MAb DHG.01 (gray) was used at a concentration of 4 μg/ml, yielding opsonic killing between 60 and 90%, and absorbed out with different amounts of native DHG (gray with vertical stripes). The purified MAb at a concentration of 4 μg/ml without inhibitor (gray) was used as the control for opsonophagocytic killing. Inhibition of the opsonophagocytic activity of anti-DHG (white) at 50 μg/ml was also performed with 8 and 4 μg/ml of native DHG (white with vertical stripes). Bars represent mean data, and the error bars represent the standard errors of the means. *, *P* < 0.05; **, *P* < 0.01; ***, *P* < 0.001; all by one-way ANOVA with Dunnett's multiple comparison to the positive control (antibody without inhibitor). Results are representative of at least two independent experiments.

The MAb DHG.01 was purified from the supernatants of the hybridomas (see Materials and Methods), and its *in vitro* activity was examined by OPA against E. faecalis type 2 at different dilutions until the activity was diminished. The MAb DHG.01 exhibited high OPK activity at concentrations ranging from 50 μg/ml to 2 μg/ml (*P* values are 0.00001 and 0.0003 for 50 μg/ml and 25 μg/ml, respectively) against the evaluated strain compared to those of the negative control of the same isotype, IgG1(κ) (Fig. 3A).

In order to examine whether the killing of the MAb DHG.01 is attributed to its specific binding to the polysaccharide, we preincubated the antibody with different concentrations of the purified polysaccharide DHG (200 μg/ml to 0.8μg/ml) prior to OPA. The OPK activity against E. faecalis type 2 was completely abolished with high concentrations of DHG and was partially restored with 4 μg/ml and 0.8 μg/ml of DHG (Fig. 3B). Purified anti-DHG was treated with 8 μg/ml and 4 μg/ml of DHG, and the same inhibition effect was observed. These results confirm not only that the MAb is opsonic but also that this activity is attributable to its binding to the polysaccharide DHG.

### SagA.01 mediates opsonophagocytosis due to its binding to SagA.

**FIG 4:**
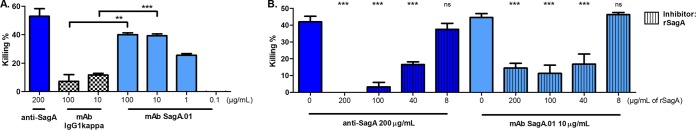
Opsonophagocytic killing activity of the purified MAb SagA.01 against E. faecium 11231/6 (A) and inhibition of its opsonophagocytic killing activity using rSagA (B). (A) The opsonophagocytic killing activity of the MAb SagA.01 was evaluated at different dilutions, ranging from 100 to 0.1 μg/ml (light blue). Polyclonal serum raised in rabbits against SagA (anti-SagA) was used as a positive control (dark blue), and a MAb of the same isotype, IgG1(κ), was used as a negative control (black and white). Bars represent mean data, and the error bars represent the standard errors of the means. **, *P* < 0.01; ***, *P* < 0.001; both by Student's *t* test compared to the negative control [MAb IgG1(κ)]. (B) The purified MAb SagA.01 (light blue) was used at a concentration of 10 μg/ml, yielding 45% killing, and inhibited with different amounts of rSagA (light blue with vertical stripes). The purified MAb at a concentration of 10 μg/ml without inhibitor (light blue) was used as a positive control for opsonophagocytic killing. As a control, purified anti-SagA at 200 μg/ml, yielding an opsonic killing of 42% (dark blue), was absorbed out with different amounts of rSagA (blue with vertical stripes). Bars represent the E. faecium mean data, and the error bars represent the standard errors of the means. ns, *P* ≥ 0.05; ***, *P* < 0.001; both by one-way ANOVA with Dunnett's multiple comparison to the positive control (antibody without inhibitor). Results are representative of at least two independent experiments.

The MAb SagA.01 was purified from supernatants of hybridomas (see Materials and Methods), and its *in vitro* activity was confirmed and more extensively examined by OPA against E. faecium 11231/6 at different concentrations, ranging from 100 μg/ml to 0.1 μg/ml. According to these results, the MAb SagA.01 exhibited high OPK activity against E. faecium 11231/6 even at 10 μg/ml. Similar OPK activity was observed at much higher concentrations for the rabbit serum raised against SagA (anti-SagA) (i.e., 200 μg/ml) (Fig. 4A). Student's *t* test demonstrated that MAb SagA.01 showed significantly more killing than the MAb of the same isotype, IgG1(κ), at the concentrations 100 μg/ml (*P* = 0.004) and 10 μg/ml (*P* = 0.000007).

In order to examine whether the killing of the MAb SagA.01 is attributable to its binding to the protein SagA on the bacterial surface, we performed an opsonophagocytic inhibition assay (OPIA). E. faecium 11231/6 was used as the target strain and recombinant SagA (rSagA) was used as the inhibitor at final concentrations ranging from 200 μg/ml to 8 μg/ml. According to our results, the OPK activity was reduced when high concentrations of rSagA were incubated with the MAb SagA.01. In addition, the OPK activity of the antibody was restored at lower concentrations of rSagA (Fig. 4B). Purified anti-SagA was treated with the same concentrations of rSagA, and the same inhibition effect was observed. These results demonstrate that the MAb is specific and its opsonic killing is attributed to the specific interaction with SagA.

### DHG.01 and SagA.01 exhibit cross-specificity toward other E. faecalis and E. faecium strains.

**FIG 5:**
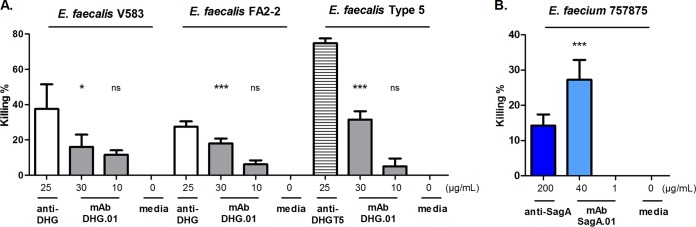
Opsonophagocytic killing activity of the MAbs DHG.01 and SagA.01 against different enterococcal strains. The opsonophagocytic killing activity of the supernatants from the hybridoma cells producing MAb DHG.01 (gray) and SagA.01 (light blue) at different dilutions was evaluated against E. faecalis V583, FA2-2, and type 5 (A) and E. faecium 757875 (B), respectively. Polyclonal sera raised in rabbits against the native polysaccharide DHG from E. faecalis type 2 (white) and E. faecalis type 5 (horizontal stripes) and SagA (dark blue) were used as positive controls. Hybridoma cell culture medium was used as a negative control. Bars represent mean data, and the error bars represent the standard errors of the means. ns, *P* ≥ 0.05; *, *P* < 0.05; **, *P* < 0.01; ***, *P* < 0.001; all by one-way ANOVA with Dunnett's multiple comparison to the negative control (media). Results are representative of at least two independent experiments.

In order to investigate the coverage of the MAb DHG.01, the supernatant from the hybridoma cells expressing DHG.01 was evaluated by OPA against different encapsulated E. faecalis strains. The strains evaluated for this purpose belonged to the E. faecalis serotypes CPS-C (i.e., E. faecalis V583 and FA2-2) and CPS-D (i.e., E. faecalis type 5), which have been found to be opsonized by anti-DHG (12). Interestingly, the MAb DHG.01 at the concentration of 30 μg/ml exhibited 16% killing against E. faecalis V583 and 18% killing against E. faecalis FA2-2. The anti-DHG serum exhibited 38% killing against E. faecalis V583 and 28% killing against E. faecalis FA2-2 in the same experiment. In addition, the MAb DHG.01 exhibited 31% opsonic killing against E. faecalis type 5, whereas the rabbit serum raised against DHG from the same strain (anti-DHGT5) exhibited 75% opsonic killing in the same experiment (Fig. 5A). For the cross-specificity of the MAb SagA.01, the supernatant from the corresponding hybridomas expressing the MAb at a concentration of 40 μg/ml was evaluated in OPA against E. faecium 757875, which has been found to be opsonized by anti-SagA (10). The supernatant exhibited 27% killing, which was twice the killing observed for the anti-SagA at a concentration of 200 μg/ml in the same experiment (Fig. 5B).

### SPR reveals similar afﬁnities of the two MAbs to their targets.

**FIG 6:**
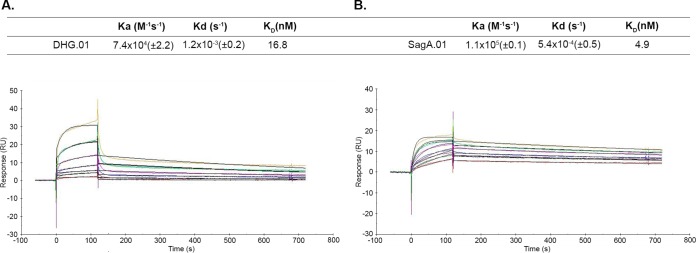
Binding kinetics and kinetic and affinity constants of DHG.01 (A) and SagA.01 (B) to DHG-SagA and SagA, respectively. Serial dilutions of the analytes DHG.01 (600 to 18.75 nM) and SagA.01 (900 to 28.1 nM) were run. The fitting model was 1:1, and the surface densities were ∼17 (DHG-SagA) and ∼30 (SagA) resonance units (RU). The numbers in parentheses represent the standard deviations of the *K_a_* and *K_d_*. Results are representative of three independent experiments.

In order to quantify the afﬁnity of the MAbs against their targets, we performed surface plasmon resonance (SPR) studies. DHG-SagA and SagA were immobilized by amino coupling on CM5 sensor chips, and the afﬁnity (*K_D_*), association (*K_a_*), and dissociation (*K_d_*) constants were determined (Fig. 6). Both DHG.01 and SagA.01 had afﬁnities in the nanomolar range (16.8 nM and 4.9 nM, respectively), which correlates with those of other antibacterial monoclonal antibodies (24, 29, 30). These results indicate that these MAbs exhibit high affinity toward their targets, which probably contributes to their activity.

### Sequencing of the monoclonal antibodies.

The heavy- and light-chain variable regions (VH and VL, respectively) of both MAbs were sequenced as described in Materials and Methods. The sequences were analyzed with IgBlast and aligned with the murine germ line sequences from which they were derived. The heavy chain of DHG.01 was derived from immunoglobulin heavy variable gene IGHV14-3*02, sharing 96.9% homology (differing in 5 amino acid residues), the diversity gene IGHD2-3*01, and the joining gene IGHJ2*01. The light chain was derived from the immunoglobulin kappa variable gene of IGKV4-90*01, sharing 98.9% homology (differing in only 2 amino acid residues) and the joining gene IGKJ2*01. According to the sequencing results, the heavy chain of SagA.01 derived from the immunoglobulin heavy variable gene IGHV3-2*02 shared 97.92% homology (differing in 6 amino acid residues), the diversity gene IGHD4-1*01, and the joining gene IGHJ2*01. The SagA.01 light chain was derived from the immunoglobulin kappa variable gene of IGKV10-96*01, sharing 98.2% homology (differing in 4 amino acid residues) and the joining gene IGKJ1*01.

### The rMAbs retain their specificity and opsonophagocytic activity.

**FIG 7:**
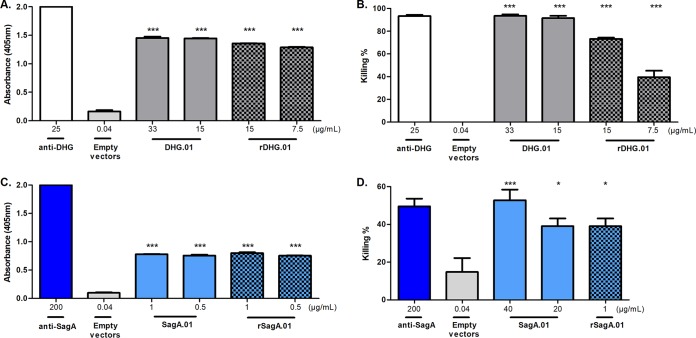
Specificity and opsonophagocytic killing activity of the MAbs rDHG.01 and rSagA.01 against E. faecalis type 2 and E. faecium 11231/6, respectively. Binding of the MAbs rDHG.01 (gray and black squares) and rSagA.01 (light blue and black squares) was evaluated by ELISA against the native DHG (A) and rSagA (C). The opsonophagocytic killing activities of the unpurified MAb rDHG.01 (gray and black squares) and rSagA.01 (light blue and black squares) was evaluated at different dilutions against E. faecalis type 2 (B) and E. faecium 11231/6 (D), respectively. Supernatants from the hybridomas expressing MAbs DHG.01 (gray) and SagA.01 (light blue) and polyclonal sera raised in rabbits against the native polysaccharide DHG (white) and SagA (dark blue) were used as positive controls. In addition, supernatant from HEK293 cells transfected with 30 μg of empty pFUSE vectors at a ratio of 2:3 (empty vectors, light gray) was used as a negative control. Bars represent the mean data, and the error bars represent the standard errors of the means. *, *P* < 0.05; **, *P* < 0.01; ***, *P* < 0.001; all by one-way ANOVA with Dunnett's multiple comparison to the negative control (empty vectors). Results are representative of at least two independent experiments.

In order to validate the obtained sequences, we constructed the full-length light and heavy chains by cloning the variable regions in the same reading frame with the constant regions in the pFUSE plasmids (see Materials and Methods). For the production of the recombinant MAbs (rMAbs), rDHG.01 (for DHG.01) and rSagA.01 (for SagA.01), HEK293 cells were transiently transfected with the combinations of the recombinant heavy and light chains. The supernatants containing rMAbs were evaluated to confirm their binding and opsonic activity. The rMAbs retained their activity in both assays, confirming the sequencing results. In particular, rDHG.01 retained 94% of its immunoreactivity in ELISA toward DHG and 80% of its OPK activity against E. faecalis type 2 compared to values for the supernatants from the hybridomas of DHG.01 when tested at a concentration of 15 μg/ml (Fig. 7A and B). Also, rSagA.01 at a concentration of 1 μg/ml retained its immunoreactivity in ELISA toward the rSagA and its OPK activity against E. faecium 11231/6 compared to those of the supernatants from the hybridomas of SagA.01 (Fig. 7C and D).

## DISCUSSION

In 2013, the Centers for Disease Control and Prevention included vancomycin-resistant enterococci (VRE) in the top 18 drug-resistant threats, a serious threat with 66,000 health care-associated enterococcal infections occurring annually in the United States, of which 1,300 are fatal (31). Cassini et al. estimated 16,146 cases of infections with VRE in the EU and European Economic Area in 2015 and an incidence of 1,081 attributable deaths (32). The resistance of these bacteria to common antibiotic therapies underlines the need for the development of new types of treatments, such as MAbs and vaccines (33). MAbs not only could serve as therapeutic agents against these multidrug-resistant bacteria but also could contribute to the rational design of vaccines (34). In particular, the elucidation of the minimal binding requirements of the protective antibodies can bring new insights in the synthesis of sugar mimetics that can be used as immunogens (34).

The high specificity of the MAbs minimizes the occurrence of unspecific binding and may therefore lead to a lower impact on the natural microbiota (35). This advantage turns to a limitation regarding the microbial coverage that a MAb may have due to the vast antigenic variability of these pathogens (36). For this purpose, our study focused on the development of MAbs against two important immunogenic antigens dominating in two groups of enterococci. Both antigens, the enterococcal protein SagA and the polysaccharide DHG, have been proven to elicit opsonic and protective antibodies against E. faecium and E. faecalis CPS-C and CPS-D (10, 12, 17, 18). Taking into account the limited number of enterococcal serotypes, these two newly developed MAbs, DHG.01 and SagA.01, combined with the human MAb that we have previously developed against lipoteichoic acid, could provide extended coverage to E. faecalis and E. faecium VRE strains (5, 37). The proposed cocktail of MAbs could overcome the antigenic variation of the pathogen that cannot be addressed with each antibody separately. However, this will be examined in more detail in future studies. Similar therapies consisting of combinations of MAbs or immunoglobulin preparations have been proposed to treat infections caused by enterococci and other Gram-positive pathogens (38–40).

The two MAbs presented in this study were developed through mouse hybridoma technology. The selection of the hybridoma cells was carried out by combining a common affinity method (ELISA) with a functional assay (OPA). The opsonophagocytic activity was examined throughout the selection process, supplementing the ELISA results and ensuring that the final clones of DHG.01 and SagA.01 retained their *in vitro* activity. To our knowledge, these two methods have not been combined before now as a selection method for the production of mouse MAbs. Our results support the utilization of this *in vitro* assay for the selection of mother-wells producing opsonic and specific antibodies and could be applied to the identification of other MAbs against different antigens and/or pathogens. In addition, the development of these two opsonic MAbs upon immunization with DHG-SagA supports our previous findings that this glycoconjugate is a good vaccine candidate against prevalent enterococcal species and that SagA could serve as a carrier protein (56).

The MAb DHG.01 exhibited high opsonic killing against E. faecalis type 2 (i.e., 90%) even at the concentration of 10 μg/ml. This activity was confirmed to be attributed to the binding of the MAb to DHG, since preincubation of the MAb with DHG inhibited its opsonophagocytic activity. In addition, the MAb SagA.01 at 10 μg/ml exhibited 40% opsonic killing, which is of great importance, since the polyclonal serum against SagA at a higher concentration exhibited 50% killing in the same assays. In a similar manner, the activity of this antibody was inhibited with rSagA, indicating the specificity of the MAb. The difference in the OPK activity of the two MAbs could be attributed to the surface availability of their targets. As previously reported, cell wall determinants could hinder the antibody recognition of the wall-associated protein vaccine candidates, leading to lower activity (17). Although the susceptibility of the bacteria to opsonophagocytosis may differ, it is noteworthy that both antibodies exhibit opsonophagocytic activity at the same concentration range as other antibacterial MAbs (24, 41, 42). Interestingly, in cross-specificity experiments the killing of the MAb DHG.01 was lower than that of the polyclonal serum raised against the native polysaccharide (Fig. 5) and to the killing of the same antibody against E. faecalis type 2 (Fig. 3A). These differences can be attributed to the high specificity of the MAb DHG.01 toward a specific epitope, which may not be equally present or exposed in all enterococcal strains. Additionally, the OPK of these MAbs remains significant compared to the OPK of the anti-DHG and anti-SagA in the same experiments. This observation confirms that both MAbs are cross-reactive against the enterococcal species possessing the two antigens.

The VH and VL of both MAbs were sequenced and reconstructed in the pFUSE expression system. After expression in HEK293 cells, the rMAbs were evaluated in ELISA and OPA. The specificity and the opsonophagocytic activity of both rMAbs were substantially retained, since in both assays minor differences between the rMAbs and the supernatants from the hybridomas expressing the MAbs were reported. The small differences observed in the activity can be attributed to the production system, for instance, small differences in the glycosylation pattern can influence the function of the antibodies (43). These results support our sequencing results but also that the *in vitro* activity that we report is solely attributable to the MAbs.

Altogether, the results presented in this study demonstrate the development of two opsonic antibodies against the polysaccharide DHG and the protein SagA. Both antibodies exhibited high opsonic killing against their bacterial targets, indicating their potential therapeutic use to treat and/or prevent enterococcal infections. Although the opsonophagocytic assay is a good indicator of the functional activity of the generated antibodies, their protective efficacy should be tested in animal models. In addition, since murine MAbs are related to immunogenicity, short half-life, and the development of human anti-mouse antibodies, the humanization of these MAbs needs to be addressed in future studies to obtain their approval for human administration (44). These antibodies ideally could replace the common antibiotic therapy but also could be used as a supplementary therapy, with antibiotics having a synergistic effect on the eradication of enterococcal infections.

## MATERIALS AND METHODS

### Bacterial strains.

In the OPAs, the prototype strain first described by Maekawa et al., E. faecalis type 2 (45), and a patient isolate of vancomycin-resistant E. faecium 11231/6 (this study) were used. For cross-reactivity tests, vancomycin-resistant bloodstream isolate E. faecalis V583 (46), patient-derived isolate E. faecalis FA2-2 (47), prototype strain E. faecalis type 5 (45), and vancomycin-resistant E. faecium 757875 (48) were used. All strains were grown in tryptic soy agar and broth (Carl Roth) without agitation at 37°C.

### Glycoconjugate production.

rSagA was overexpressed and purified as described previously (17). Enterococcal polysaccharide DHG, previously purified as described by Theilacker et al. (12), was covalently coupled to rSagA using organic cyanylating agent 1-cyano-4-dimethylaminopyridinium tetrafluoroborate (CDAP) as described by Lees et al. (49). In particular, a fresh solution of CDAP (Sigma-Aldrich) at 100 mg/ml in acetonitrile was prepared. Two mg of DHG was diluted in 200 μl of ultrapure water, and 20 μl of CDAP (100 mg/ml) was slowly added to the vortexed solution of the polysaccharide. After 30 s of vortexing, 30 μl of 0.2 M trimethylamine was added. At 2.5 min of vortexing, 2 mg of protein in 0.25 M HEPES buffer (pH 8.4) was added to the mixture, and the reaction mixture was incubated overnight at room temperature with shaking. The resulting glycoconjugate, DHG-SagA, was washed with phosphate-buffered saline (PBS) supplemented with 250 mM NaCl and cleaned up with successive washes with PBS using a 100-kDa Amicon Ultra centrifugal filter (Merck-Millipore). Finally, the conjugate was sterile filtered using 0.20-μm Spin X centrifuge tube filters (Corning-Costar). The conjugation process was evaluated by SDS-PAGE, Stains All staining, and Western blotting. Finally, protein and sugar content were determined by Bradford and Anthrone assays, respectively (50, 51), and the final sugar-to-protein ratio was calculated to be 1 to 4.

### Rabbit immunizations.

New Zealand White rabbits immunized with purified DHG from either E. faecalis type 2 (anti-DHG) or E. faecalis type 5 (anti-DHGT5) and SagA (anti-SagA) are described elsewhere (10, 12). These sera were used as positive controls in all assays. Sera were purified by using rProtein A GraviTrap columns (GE Healthcare) by following the manufacturer’s instructions.

### MAb generation.

BALB/c mice were immunized by two subcutaneous injections of 50 μg, in protein content, of the glycoconjugate DHG-SagA with Freund's incomplete adjuvant given 2 weeks apart. After the second immunization, the mouse sera were screened by ELISA for antibody titers against the immunogen. Three weeks later, a final intraperitoneal injection of 50 μg of glycoconjugate in PBS was given to the mouse with the highest titers. Three days after the injection, the splenocytes were isolated and fused with SP2/O myeloma cells at a 1:1 ratio after elimination of the red blood cells by lysis. The cells were once resuspended in RPMI supplemented with 20% fetal bovine serum (FBS) (PAN-Biotech) containing hypoxanthine, aminopterin, and thymidine (Gibco) for the selection of the hybridomas and were equally distributed into six 96-well plates. The supernatants from the mother-wells were screened by ELISA against the immunogens and by OPA against the target bacterial strains. The mother-wells that exhibited immunospecificity to the targets were subjected to cloning by limiting dilution to obtain monoclonal cell populations. The obtained cell lines were propagated and maintained in 10% FBS, 2 mM l-glutamine, 10 mM HEPES, and RPMI 1640 (Gibco).

For the MAb purification, hybridoma cells were grown in ISF-1 medium (Biochrom), and their supernatants were collected and purified by protein G affinity chromatography using an ÄKTA pure chromatography system (GE Healthcare). For the OPAs, a mouse MAb of the same isotype, IgG1(κ), which has no immunoreactivity to several bacterial antigens, was selected from our collection as a negative control. This MAb was produced and purified from the hybridomas under the same conditions as those for the rest of the evaluated MAbs.

### ELISA.

Nunc-Immuno MaxiSorp MicroWell 96-well plates were coated with DHG or rSagA (1 μg/well) in coating buffer (0.2 M carbonate-bicarbonate) and incubated overnight at 4°C. The plates were washed three times with washing buffer (WB; PBS, 0.05% Tween 20) and blocked with blocking buffer (BB; 3% bovine serum albumin [BSA] in PBS) for 1 h at 37°C. After plates were washed two times with WB, serial dilutions of the supernatants from the hybridomas or the control sera in BB were plated in triplicates. After 1 h of incubation, the plates were washed three times with WB, and the secondary antibody, alkaline-phosphatase-conjugated anti-mouse or anti-rabbit IgG produced in goat (Sigma-Aldrich), at 1:1,000 dilution, was added. The plates were incubated for 1 h at room temperature (RT) and washed three times with WB, and the detection was performed using 1 mg/ml *p*-nitrophenyl phosphate (Sigma-Aldrich) in glycine buffer. After 30 min of incubation at RT, absorbance was measured at 405 nm in a Synergy H1 hybrid reader (BioTek). For the selection of the clones and the determination of the isotype of the MAbs, plates were coated using MAbs specific to the different isotypes of the heavy (IgG1, IgG2a, IgG2b, IgG2c, IgG3, and IgM) (Jackson ImmunoResearch) and light (kappa and lambda) (AbD Serotec) chains, and horseradish-peroxidase-conjugated anti-mouse IgG and IgM were used as secondary antibodies (Jackson ImmunoResearch). The detection was performed using *o*-phenylenediamine dihydrochloride in citrate buffer supplemented with 0.12% H_2_O_2_. After incubation for 5 to 10 min in the dark, the reaction was stopped with 1 M H_2_SO_4_ and absorbance was measured at 492 nm and 630 nm in a TriStar LB941 reader (Berthold).

### Western blotting.

The glycoconjugate DHG-SagA and rSagA were analyzed by SDS-PAGE using 4 to 12% Bis-Tris protein gels in morpholineethanesulfonic acid SDS running buffer (NuPAGE Novex; Invitrogen). Electrophoresis was run for 45 min at 170 V in an XCell SureLock minicell (Invitrogen), and immunoblotting was performed at RT for 1 h at 30 V in an XCell II blot module (Invitrogen) using polyvinylidene difluoride membranes (0.2-μm pore size; NuPAGE Novex; Invitrogen). Membranes were blocked with BB overnight at 4°C. The next day, they were washed three times with WB and incubated for 1 h with 2 μg/ml of MAb in BB. The membranes then were washed again as described above and incubated for 1 h with alkaline phosphatase-conjugated anti-mouse IgG (Sigma-Aldrich) diluted 1:1,000 in BB. Finally, three washes with WB were performed and binding was detected by the colorimetric AP substrate reagent kit (Bio-Rad).

### OPA and OPIA.

MAb activity was evaluated by OPA as previously described (10) using the bacterial strain, baby rabbit complement, white blood cells (WBCs), and purified MAbs or supernatants from the hybridoma cells without antibiotics. Bacteria were grown at 37°C until the optical density at 650 nm reached 0.4 and adjusted to a final concentration of 2 × 10^7^ CFU/ml in RPMI 1640 (Gibco) with 15% FBS (termed 15% RPMI). Rabbit complement (Cedarlane) was diluted at a final concentration of 6.7%, vol/vol, in 15% RPMI, incubated with the target strain for 60 min at 4°C with shaking, and filter sterilized. WBCs, freshly isolated from a healthy human donor, were prepared by mixing blood with an equal volume of heparin-dextran buffer. After incubation for 45 min at 37°C, the upper layer was collected and centrifuged (at 2,700 rpm for 10 min at 10°C), and the resulting pellet was washed with 15% RPMI. The erythrocytes in the pellet were lysed with 1% NH_4_Cl (Sigma-Aldrich) at RT for 20 min. WBCs were washed again and resuspended in 15% RPMI to yield a final concentration of ∼2 × 10^7^cells/ml. The four components were added in equal volumes and incubated on a rotor rack at 37°C for 90 min. The samples were plated in quadruplicates to enumerate the CFU. The percentage of killing was calculated by comparing the surviving CFU in the reaction WBCs (WBCpos) to the surviving CFU in the tubes lacking WBCs (WBCneg) using the following formula: % killing = 100 − [100 × (WBCpos mean CFU at 90 min)/(WBCneg mean CFU at 90 min)]. Negative controls lacking one, two, or three of the components were included in the assay. In all experiments presented, no killing was observed for these four negative controls. For the OPIAs, the MAbs and the rabbit sera were preincubated with different concentrations of rSagA or DHG overnight at 4°C with shaking. OPA was performed as described previously, using this mixture as an antibody source. The levels of inhibition were compared to that of a negative control having the same concentration of MAb incubated overnight at 4°C but the protein or the polysaccharide inhibitor replaced by PBS or water, respectively.

### SPR.

Binding kinetics and affinities were determined by SPR using a Biacore X100 system as described elsewhere (52). Amine-coupling immobilizations were performed on CM5 sensor chips (Biacore) using 0.5 μg/ml of conjugate or protein in 20 mM sodium acetate, pH 4. The surface densities obtained were ∼17 and 30 resonance units for DHG-SagA and SagA, respectively. Sensorgram data were analyzed using BIAevaluation software (Biacore).

### Antibody sequencing.

Total RNA was isolated from 5 × 10^6^ hybridoma cells using the RNeasy kit (Qiagen) and reverse transcribed into cDNA using the SuperScript III first-strand synthesis system for reverse transcription PCR (Invitrogen) by following the manufacturer’s instructions. PCR was performed with GoTaq hot start green master mix (Promega) using the cDNA as the template. The sense primers for the VH and VL regions anneal to the leader sequence, whereas the VH and VL antisense primers anneal to the IgG1 heavy-chain and immunoglobulin kappa light-chain constant regions, respectively (Table 1). The PCR products were purified with Wizard SV gel and PCR clean-up system (Promega) and cloned into the TOPO TA cloning vector pCR2.1 (Invitrogen) by following the manufacturer’s instructions. After cloning, plasmids were purified using the Wizard plus SV minipreps DNA purification system (Promega) and sequenced by Eurofins Scientific (Germany). The VH and VL of SagA.01 were sequenced by GenScript. The resulting sequences were analyzed using NCBI IgBLAST (https://www.ncbi.nlm.nih.gov/igblast).

**TABLE 1:** Primers used for amplification of variable domains

Name	Primer sequence	Reference or source
VH		
Mouse_IGG1_Fw_P1	ATGGAATGCAGCTGGGTCATCCTCTT	54
Mouse_IGG1_Fw_P2	ATGGGATGGAGCTGTGTAATGCTCTT	54
Mouse_IGG1_Fw_P3	ATGAACTTCGGGCTGAGCTTGATTTT	54
Mouse_IGG1_Fw_P4	ATGGCTGTCTTGGGGCTGCTCTTCT	54
Mouse_IgG1_deg_Fw_1	ATGRASTTSKGGYTMARCTKGRTT	55
Mouse_IgG1_deg_Fw_2	ATGRAATGSASCTGGGTYWTYCTCT	55
Mouse_IGG1_Rv_Cons	CAGGGGCCAGTGGATAGAC	This study
VL		
Mouse_Kappa_Fw_P9	ATGGAGACAGACACACTCCTGCTAT	54
Mouse_Kappa_Fw_P10	ATGGATTTTCAAGTGCAGATTTTCAG	54
Mouse_Kappa_Fw_P11	ATGGAGTCACAGACTCAGGTCTTTATA	54
Mouse_Kappa_Fw_P12	ATGGCCCCAACTCAGCTCCTGGT	54
Mouse_Kappa_Fw_P13	ATGAAGTTGCCTGTTAGGCTGTTG	54
Mouse_Kappa_Rv_Cons	TTAACACTCATTCCTGTTGAAGC	This study

### rMAb construction.

rMAbs were constructed using the pFUSE-CHIg-mG1 and pFUSE2ss-CLIg-mK expression system (InvivoGen). The VHs were amplified by PCR using the primer HP_Rv_pFUSE combined with either the primer DHG.01_HP1_Fw_pFUSE or the primer SagA.01_HP_Fw_pFUSE, whereas the VLs were amplified using the primer KP_Rv_pFUSE combined with either the primer DHG.01_KP10_Fw_IL2_signal or the primer SagA.01_1K_Fw_IL2_SP (Table 2). After digestion with the corresponding restriction enzymes (NEB), the VHs were cloned into the vector pFUSE-CHIg-mG1, whereas the VLs were cloned into the vector pFUSE2ss-CLIg-mK. The constructs were electroporated into the E. coli TOP10 cells, and midi preps were prepared using a Plasmid plus midi kit (Qiagen).

**TABLE 2:** Primers used for reconstruction of rMAbs

Name	Primer sequence^*a*^	Source
VH		
DHG.01_HP1_Fw_pFUSE	GACCGGCGCCTACCTGAGATCACCGGTATGGAATGCAGCTGGGTCATC	This study
SagA.01_HP_Fw_pFUSE	GACCGGCGCCTACCTGAGATCACCGGTATGAGAGTGCTGATTCTTTTG	This study
HP_Rv_pFUSE	TCGTTTTAGCGCTGGAGACT	This study
VL		
DHG.01_KP10_Fw_IL2_signal	GCGCGAATTCAGAAATTTTGCTCACCCAGTC	This study
SagA.01_1K_Fw_IL2_SP	GCGCGAATTCAGATATCCAGATGACACAGAC	This study
KP_Rv_pFUSE	CGTTTTATCTCGAGCTTGGTC	This study

a The KasI, AfeI, EcoRI, and XhoI restriction sites are underlined.

### Transient production in HEK293 cells.

Plasmid combinations of the corresponding VL and VH were cotransfected in HEK293 cells as described elsewhere (53). Briefly, cells were seeded in 15% Dulbecco’s modified Eagle’s medium plus GlutaMAX (Gibco) in a TC dish 150 (Sarstedt). The next day, 1 h prior to the transfection, the medium was replaced with Opti-MEM reduced-serum medium supplemented with GlutaMAX (Gibco), and then 120 μg of polyethylenimine MW25000 (Polysciences) was added with 30 μg of the plasmids using the VH and VL vectors at a 2:3 ratio. After 24 h, the medium was changed for RPMI supplemented with 2% ultralow IgG FBS (Gibco). Supernatants from the transfected HEK293 cells were harvested after 4 days, quantified (see the supplemental material), and examined by ELISA and OPA.

### Statistical analysis.

Statistical analysis was performed using the software GraphPad PRISM, version 5.00, using one-way analysis of variance (ANOVA) with Dunnett's multiple comparison or Student's *t* test, as indicated. The data were expressed as the geometric means ± standard errors of the means.

### Ethics statement.

Mouse experiments were approved by the Ethics Committee of the Veterinary Department of the Ministry of Agriculture and Animal Welfare Committee of the University of Rijeka, Faculty of Medicine. This study was carried out in accordance with the recommendations of regulations on the protection of animals used for scientific purposes (Official Gazette of the Republic of Croatia, 55/2013).

## Supplementary Material

Supplemental file 1
