# Supplementary material for: Development of Opsonic Mouse Monoclonal Antibodies against Multidrug-Resistant Enterococci
Source: Infect Immun. 2019 Aug 21;87(9):e00276-19. doi: 10.1128/IAI.00276-19 (PMC6704603; doi:10.1128/IAI.00276-19)
Supplement: Supplemental file 1 [file IAI.00276-19-s0001.pdf]

SUPPLEMENTARY MATERIALS AND METHODS

Quantification of mouse IgG concentrations

The mAb concentration in the supernatants from hybridomas, as well as the concentrations from all rabbit sera were determined by sandwich ELISA as previously described by Salauze et al. (1). Nunc-immuno Maxisorp MicroWell 96 well plates were coated with 0.1µg per well of unlabeled a-mouse IgG antibody (SouthernBiotech) or a-rabbit IgG antibody (Sigma-Aldrich) in coating buffer. The plates were incubated overnight at 4°C, wells were washed three times with washing buffer IgWB (0.9% sodium chloride, 0.1% Tween 20) and incubated with BB for 1hour at RT. After blocking, wells were washed three times with IgWB and serial dilutions in BB of either the supernatants from the hybridoma cells or the rabbit sera were plated in triplicates. Meanwhile, dilutions in BB of either standard mouse IgG (SouthernBiotech) or rabbit IgG ranging from 31.2ng/mL to 0.24ng/mL were plated in triplicates. After 2hour incubation, wells were washed three times with IgWB and the secondary antibody, AP conjugated a-mouse or a-rabbit IgG produced in goat (Sigma-Aldrich), at 1:1000 dilution was added. The incubation was carried out for 2hours at RT, the wells were washed four times with IgWB and detection was performed using p-nitrophenyl phosphate at 1mg/mL in glycine buffer. After 30min of incubation at RT the absorbance was measured at 405nm. For the calculation of the antibody concentration the calibration curves of the standard mouse or rabbit IgG dilutions were used.

References

1. Salauze D, Serre V, Perrin C. 1994. Quantification of total IgM and IgG levels in rat sera by a sandwich ELISA technique. Comp Haematol Int 4:30–33.
